# Thyroid hormone action during GABAergic neuron maturation: The quest for mechanisms

**DOI:** 10.3389/fendo.2023.1256877

**Published:** 2023-10-03

**Authors:** Sabine Richard, Juan Ren, Frédéric Flamant

**Affiliations:** Institut de Génomique Fonctionnelle de Lyon, UMR5242, Ecole Normale Supérieure de Lyon, Centre National de la Recherche Scientifique, Université Claude Bernard-Lyon 1, USC1370 Institut National de Recherche pour l’Agriculture, l’Alimentation et l’Environnement, Lyon, France

**Keywords:** brain development, parvalbumin interneurons, thyroid hormone receptors, animal models, hypothyroidism, neurological disorders

## Abstract

Thyroid hormone (TH) signaling plays a major role in mammalian brain development. Data obtained in the past years in animal models have pinpointed GABAergic neurons as a major target of TH signaling during development, which opens up new perspectives to further investigate the mechanisms by which TH affects brain development. The aim of the present review is to gather the available information about the involvement of TH in the maturation of GABAergic neurons. After giving an overview of the kinds of neurological disorders that may arise from disruption of TH signaling during brain development in humans, we will take a historical perspective to show how rodent models of hypothyroidism have gradually pointed to GABAergic neurons as a main target of TH signaling during brain development. The third part of this review underscores the challenges that are encountered when conducting gene expression studies to investigate the molecular mechanisms that are at play downstream of TH receptors during brain development. Unravelling the mechanisms of action of TH in the developing brain should help make progress in the prevention and treatment of several neurological disorders, including autism and epilepsy.

## 1 Introduction

Thyroid hormone (TH) signaling plays a major role in mammalian brain development (1). Any alteration in TH economy during brain development – be it TH synthesis, TH transport or activity of TH receptors (TRs) – is likely to induce long-lasting and irreversible defects, ranging from mild intellectual disability to profound physical and mental impairments. This has been known for a long time, but the precise underlying mechanisms are still unknown. Data obtained in the past years in animal models have pinpointed the GABAergic system as a major target of TH signaling during development (2, 3). GABAergic neurons, which use GABA (gamma-aminobutyric acid) as a neurotransmitter, are the chief inhibitory neurons in the vertebrate central nervous system. Alteration in the development of the GABAergic system is known to be associated with neurological disorders such as intellectual disability, autism spectrum disorder (ASD), and epilepsy (4). On the other hand, thyroid dysfunction is often associated with some of these neurological disorders, such as anxiety and seizure susceptibility (5, 6). Wiens and Trudeau (7) have previously reviewed *in vitro* and *in vivo* evidence in rodents, indicating that alterations in TH signaling may affect the GABAergic system in several ways: GABA synthesis and metabolism, GABA release and reuptake, GABA receptor expression and function, etc. However, the molecular and cellular mechanisms by which TH impacts GABAergic neurons are unknown. The aim of the present review is to gather the available information about the direct involvement of TH in GABAergic neuron maturation.

TH (either its most active form T3: 3,3’,5-triiodo-L-thyronine, or its less active precursor T4: thyroxine) binds to nuclear receptors, which are transcription factors regulating gene expression. Two genes (*THRA* and *THRB* in humans, *Thra* and *Thrb* in rodents) encode the TRα1, TRβ1 and TRβ2 nuclear receptors. While TRα1 is present at all developmental stages in many cell types and in all the rodent brain areas, TRβ1 mRNA appears later and TRβ2 is restricted to few brain areas (8). However, alternate splicing of *Thra* mRNA also generates the TRα2 mRNA, which encodes a non-receptor protein. Notably, in the brain, TRα2 is more abundant than TRα1 (9). Knowing that direct measurement of TR protein concentrations is difficult, due to poor antibody specificity and very low abundance of TR proteins, the respective abundance of the different TR isotypes in the different cell types remains unclear. In humans, old studies of TH binding suggest that TRβ1 is the predominant receptor in the human fetal brain (10).

More convincingly than TR expression patterns, both human and mouse genetics clearly demonstrate that TRα1 mediates crucial, significant actions of TH on early brain development, while TRβ1 and TRβ2 appear to be necessary for more specific and discreet steps of brain development (11–14). Patients who bear mutations in *THRA* are very likely to present significant neurological disorders such as epilepsy, motor incoordination or impaired cognitive function (15, 16). *THRB* mutations alter the regulation of the hypothalamo-pituitary-thyroid axis, increasing the circulating level of TH, which may in turn alter neurodevelopment. Neurocognitive impairment has been frequently associated with mutations in *THRB*, but the consequences on brain function appear to be less dramatic than those induced by *THRA* mutations (17, 18).

In the present paper, we will retrace how rodent models have helped uncover the critical role of TH/TR signaling in the differentiation of GABAergic neurons. Deciphering the network of TH/TR target genes in GABAergic neurons during brain development appears as a promising way of generating novel approaches to alleviate various neurodevelopmental disorders associated with GABAergic dysfunction, including epilepsy and ASD. Moreover, identifying TH target genes in GABAergic neurons during brain development is likely to contribute to a more general understanding of TH action in the brain.

## 2 Disruption of TH signaling during development induces a wide array of neurological disorders, including GABAergic dysfunction

Disruption of TH signaling may arise from very different causes affecting various aspects of TH molecular landscape (19). In mammals, TH is synthesized in the thyroid gland from tyrosine and iodine. In the blood and cerebrospinal fluid, TH is partly bound to distributor proteins, such as transthyretin, thyroxine-binding globulin, albumin and lipoproteins (20). The main source of TH for the fetus is from the mother throughout gestation, since even when the fetal thyroid starts to synthesize TH, the maternal TH remains the main source of circulating fetal TH (21). Transfer of maternal TH to the fetal brain involves crossing the placental and blood-brain barriers. TH entry into the brain is facilitated by specific transmembrane transporters, with monocarboxylate transporter 8 (Mct8) and organic anion transporter polypeptide 1c1 (Oatp1c1) playing a prominent role in TH brain transport in rodents (1). Around 80% of the T3 that is present in the brain derives from local deiodination of T4, mediated by type 2 iodothyronine deiodinase (Dio2), while the rest originates from the circulation (22). Deiodinase 3 (Dio3) degrades T4 and T3 into inactive metabolites and is thus the major physiological TH inactivator (23). As a consequence of the multiplicity of molecular actors involved from maternal TH synthesis to regulation of the expression of TH target genes, a wide variety of causes may affect thyroid hormone economy in the fetal brain (24): iodine deficiency, insufficient TH synthesis by the thyroid gland, problems in blood and cerebrospinal fluid transport of TH, abnormalities in the placental barrier, defects in membrane transport proteins, altered enzymatic activity of deiodinases, defects in cytosolic TH binding proteins, and last but not least, defects in TH receptor activity. The consequences of such defects have been previously described in the specialized literature. From this array of causes, TH deficiency during fetal and postnatal development may cause diverse kinds of neurological impairment (1). As a matter of example, we have chosen to mention here three emblematic diseases linked to poor TH signaling during development, one linked to a lack of hormone, the second to a deficit in TH membrane transporters and the third to a deficit in TH receptors.

- Congenital hypothyroidism, defined in humans as partial or complete TH deficiency at birth, can start early during development and represents the major cause of preventable intellectual disability in the world. If not detected and treated early, congenital hypothyroidism can have devastating effects on neurocognitive function. The clinical spectrum goes from mere developmental delay to persistent intellectual disability, hearing and speech impairment, psychomotor impairment, with the most severe condition being historically known as cretinism (25, 26). A major factor influencing the degree of severity of the symptoms is the time at which TH deficiency occurs, relative to brain developmental steps. In children with normal thyroid function born to hypothyroid mothers, TH starts to be synthesized shortly before birth, which allows partial recuperation after the initial developmental delay. In hypothyroid children born to euthyroid mothers, the maternal TH contribution during the latter part of gestation also provides partial compensation for the inadequate fetal TH supply. By contrast, hypothyroidism that stems *in utero* and that extends throughout childhood has more severe consequences (27). Newborn screening, accompanied by T4 replacement therapy, has been efficiently implemented for decades in several countries, but most newborns worldwide remain away from such protocols of screening and treatment (28). Moreover, even under T4 treatment, significant impairment in clinical and cognitive scores may persist in children with congenital hypothyroidism (29).
- Allan-Herndon-Dudley syndrome is a rare X-linked disease that affects human males with mutations in the *Slc16a2* gene encoding Mct8, which is critically needed for TH to enter the human brain. Allan-Herndon-Dudley patients have a shortened life expectancy and present with physical and intellectual disability, speech deficits and severe neurological abnormalities, including, in some cases, epilepsy (30–33). Histopathological analyses have revealed that hypomyelination is the most salient feature of the brains of Allan-Herndon-Dudley patients. Notably, at the level of the cerebral cortex, López-Espíndola et al. (34) have shown that this syndrome is associated with a reduction in the numbers of parvalbumin (PV)-expressing neurons, a category of GABAergic interneurons which play pivotal roles in cortical development and function. In addition, Allan-Herndon-Dudley patients’ cerebellum displays abnormal differentiation of Purkinje cells, which are GABAergic projection neurons (34).
- Resistance to thyroid hormone receptor alpha (RTHα) is another rare disease due to mutations in *THRA*, the gene coding for TRα1. The clinical features of patients with RTHα are quite heterogeneous. Delayed milestones in the development of motor and speech abilities are the most common neurological symptoms (15). Notably, among the 40 reported cases to date, three had epilepsy (35–37), which significantly outweighs the incidence of epilepsy in the general population [4-10 per 1,000 people (38)]. Moreover, it has been suggested that the proportion of *THRA* mutations was higher in ASD patients than in the general population. Testing this possibility, Kalikiri and colleagues (39) made the astonishing discovery of seven novel *THRA* mutations, all likely to be pathological, in a small cohort of 30 patients with ASD in India. This adds to another case of ASD with a *THRA* mutation, which was previously discovered in Canada (40).

The variety of neurological symptoms associated with TH signaling defects is explained by the fact that TH influences a wide panel of cellular processes in the developing brain, such as neurogenesis, neuronal migration, neuronal and glial cell differentiation, myelination and synaptogenesis (1). Accordingly, many genes have been found to be under direct or indirect regulation by TH (41), and deciphering the precise mechanisms underlying TH action will necessarily involve isolating direct from indirect effects.

## 3 Rodent models point to the GABAergic system as a major target of TH/TR signaling during development

The timing of neurodevelopmental stages differs significantly between humans and rodents, rodent early post-natal stages roughly corresponding to the end of the second trimester of human pregnancy (24). However, as major steps in brain development are conserved between humans and rodents, our understanding of the role of TH in the developing brain has greatly benefited from rodent studies. Seminal work undertaken to decipher the actions of TH in the developing brain involved rat models of congenital hypothyroidism. In the past decades, genetic tools have allowed to develop mouse models precisely designed to dissect the effects of TH in specific cells of the brain. The following sections will focus on these models and their contribution to our understanding of the role of TH in GABAergic neuron development.

### 3.2 Rat models of congenital hypothyroidism show defects in GABAergic neuron maturation

The morphological consequences of congenital hypothyroidism have been extensively studied and include alterations in cortical lamination, high density of hippocampal neurons, poor differentiation of the gray-white matter boundary and delayed cerebellar development (1). The following paragraphs will focus on the effects of developmental hypothyroidism on GABAergic neurons.

#### 3.1.1 Cerebellum

The relative simplicity of the microanatomy of the cerebellar cortex, as well as its strong TH signaling dependency, make it an excellent model to study the neurodevelopmental function of TH. The proliferation and migration of granule cells, which represent the vast majority of the neuronal population in the cerebellum, are stimulated by contacts with a monolayer of GABAergic projection neurons, called Purkinje cells. Congenital hypothyroidism dramatically affects the morphological maturation of Purkinje cells. The growth, dendrite arborization and dendrite spine number of Purkinje cells are markedly decreased in hypothyroid rats (42–44). The cerebellum also contains GABAergic interneurons, called basket, stellate and Golgi cells. Early studies of rat cerebellum have shown that congenital hypothyroidism delayed the postnatal increase in GABA receptor density (45), and lowered the final number of basket cells (42). More recently, Manzano et al. (46) have reported that hypothyroid rats at postnatal day (PND) 16 exhibited a decreased number of Golgi cells, as well as a delayed disappearance of the precursors of cerebellar GABAergic interneurons. Moreover, they found that on PND8, the proliferation of GABAergic interneuron precursors in cerebellar white matter was reduced in hypothyroid rats. Thus, several components of the GABAergic system are impaired in the hypothyroid rat cerebellum.

#### 3.1.2 Cortex and hippocampus

In 1996, Berbel et al. (47) were the first to describe the impact of severe congenital hypothyroidism on a subset of cortical GABAergic inhibitory neurons expressing the calcium-binding protein parvalbumin (PV). They described a striking reduction in PV-positive terminals in the neocortex of adult hypothyroid rats (47). Ten years later, Gilbert et al. (48) further showed that moderate degrees of TH insufficiency during development were sufficient to induce a significant reduction of PV fiber staining and PV cell body count in rat cortex and hippocampus at PND23. By contrast, hypothyroid rats have been shown to exhibit an increased density of calretinin neurons, another GABAergic interneuron subtype, in the dentate gyrus of the hippocampus (48). These effects were in part irreversible, since returning to a euthyroid state in adulthood only allowed partial recovery (48, 49). By means of cross-fostering and hormonal replacement studies, Gilbert et al. (48) also emphasized that the developmental window over which TH insufficiency occurred was determinant, the first postnatal weeks appearing as the most critical stages for TH influence on PV expression in the cortex and hippocampus. However, TH insufficiency that spanned the prenatal and postnatal period produced more profound deficits in PV staining than postnatal insufficiency alone. Of note, adult-onset hypothyroidism did not appear to impact the expression of PV in the cortex and hippocampus (48). Last but not least, the number of GABAergic neurons in the cortex and hippocampus was not altered by congenital hypothyroidism, indicating that the decrease in PV staining resulted from an alteration in phenotypic expression of PV, rather than neuronal loss (48). This was later confirmed by showing that hypothyroid rats did not differ from controls in the number of cells that expressed a GABA-synthesizing enzyme, GAD67 [glutamic acid decarboxylase 67 aka GAD1; (50)]. Intriguingly though, the expression of another GAD isotype, GAD65 (aka GAD2), was significantly reduced in both neuronal somata and processes in the hippocampus of the same hypothyroid rats (50). In a separate study, it was also found that the protein levels of GAD67 were lower in the medial prefrontal cortex of hypothyroid, compared to control, rats (51). In rats, but not in mice (52), a defect in neuronal migration causes heterotopia, i.e. the accumulation of gray matter in the corpus callosum (53). Of note, a few GABAergic neurons have been identified within the heterotopia, even though they constitute a minority of the heterotopic cells.

Electrophysiology studies of the dentate gyrus of the hippocampus in adult rats that were hypothyroid during development, have revealed that alterations in GABAergic interneuron populations were associated with functional deficits in inhibitory synaptic transmission (48, 54). Accordingly, in the hippocampus of hypothyroid rats at PND15, there was a near 80% reduction in KCC2 protein, a neuron-specific K^+^/Cl^-^ cotransporter that is a key player in determining the response of excitatory neurons to GABAergic neurotransmission (50).

### 3.2 Genetically-modified mouse models shed light on the molecular mechanisms underlying TH action on developing GABAergic neurons

Mutations of proteins of the TH signaling pathway, notably TRs and TH transporters, often cause defects in GABAergic neuron differentiation in the cerebellum, cortex, hippocampus and other brain regions (**Table 1**).

**Table 1:** Mouse models with gene mutations that are cited in the present paper.

Protein	Protein function	Type of mutation	Floxed	Mouse Genome Informatics reference*	Alias	GABAergic phenotype
Type 2 deiodinase	Conversion of T4 into T3	KO		Dio2^tm1Acb^	*Dio2 KO*	Weak
Type 3 deiodinase	Conversion of T4 and T3 into inactive metabolites	KO		Dio3^tm1Stg^	*Dio3 KO*	Weak
Oatp1c1	TH transporters	KO	Yes	Slco1c1^tm1Arte^	*Oatp1c1fl*	No
Mct8		KO		Slc16a2^tm1a(KOMP)Wtsi^	*Mct8 KO*	No
		KO		Slc16a2^tm1Dgen^	*Mct8 KO*	No
		KO		No MGI reference. See Wirth et al. *J. Neurosci.*, July 29, 2009, 29(30):9439 –9449	*Mct8-*	No
		KO		Slc16a2^tm1.1Sref^	*Mct8 KO*	Yes
		KO	Yes	Slc16a2^tm1c(KOMP)Wtsi^	*Mct8fl*	Yes
		Missense KI (P253L)		Slc16a2^em2Agfz^	*P253L*	Yes
TRα1	Nuclear receptor of T3	Frameshift KI		Thra^em1Ffla^ to Thra^em4Ffla^	*Thra^S1,S2,L1,L2^*	Yes
		Frameshift KI	Yes	Thra^em6Ffla^	*Thra^Slox^*	Yes
		KO		Thra^tm1Ven^	*TRα1^KO^*	No
		KO		Thra^tm2Jas^	*TRα^0^*	No
		KO		Thra^tm1Jas^	*TRα-*	No
		Missense KI (L400R)	Yes	Thra^tm1Ffla^	*Thra^AMI^*	Yes
		Missense KI (R384C)		Thra^tm3Ven^	*TRα1^m^*	Yes
TRβ1 TRβ2	Nuclear receptors of T3	KO		Thrb^tm1Df^	*Thrb KO-*	No

* https://www.informatics.jax.org/

All these models were used to analyze the function of thyroid hormone in mouse brain development, sometimes in combination with one another.

In several instances, it was found that mutant mice expressing a *Thra* or *Thrb* knock-in mutation exhibited stronger phenotypes than mice in which *Thra* or *Thrb* had been knocked out. This results from a number of reasons, notably that (12)some of the knock-in mutated alleles encoding for TRs exert dominant-negative activity: they prevent the normal function of intact TRs that are still present in cells. Therefore, some germline *Thra* knock-in mutations have particularly drastic effects on brain development, even in heterozygous mice (55). Indeed, dominant-negative receptors constitutively interact with corepressor proteins, and thus permanently repress the expression of TR target genes, whether TH is present or not. Moreover, it has been observed that *Thra* knock-in mutations, encoding for dominant-negative forms of TRα1, do not only affect the transcription of TRα1 target genes, but also induce a repression of known TRβ1 target genes, thus strengthening the impact on the resulting phenotype (56). However, the main explanation for the mild neurological consequences of KOs is that getting rid of TRs does not only eliminate TH-induced activation of gene expression, but also eliminates the transcriptional repression mediated by unliganded or mutant receptors (12, 57).

#### 3.2.1 Cerebellum

Mice with a dominant-negative *Thrb* allele exhibit severe neurological deficiencies, notably a marked impairment in balance and coordination, and profound defects in cerebellar development, notably in the number and arborization of cerebellar Purkinje cells (58, 59). By contrast, no reduction in Purkinje cell number was found in *Thrb* KO mice, which appeared to exhibit normal neurological development, with the exception of a loss of auditory function (60, 61).

In the cerebellum of TRα1 KO mice, there was no apparent defect in granule cell migration, nor in Purkinje cell morphology (12), but further analyses revealed a reduced number of GABAergic interneuron precursors between PND4 and PND10, a reduced rate of proliferation of GABAergic interneuron precursors in the white matter at PND6 as well as a reduced expression of a GABAergic transporter (GAT-1) at PND11 (46)

The development of the cerebellum was also found to be significantly impaired in *Dio3^-/-^* mice, in which the intracellular TH content is increased (62). Notably, *Dio3^-/-^* mice exhibited accelerated expansion of the molecular layer, which contains the dendritic tree of Purkinje cells. However, the number of Purkinje cells did not differ between *Dio3^-/-^* and control mice. Notably, the expansion of the molecular layer follows a normal timing in *Dio3^-/-^ TRα1^-/-^* double KO (DKO) mice, indicating a role for TRα1 in mediating the action of TH on Purkinje cell maturation.

In knock-in mice expressing TRα1^R384C^, which are characterized by a 10-fold reduction in the affinity of TRα1 to TH, there was an overall delay in the development of the cerebellum. Cerebellar Purkinje cells showed a delayed, but otherwise normal, arborization (2). At PND9, the expression of PV, calbindin and calretinin was lower in mice expressing TRα1^R384C^ than in control mice, but these differences were normalized a few days later (63). By PND21, the structure of the cerebellum was similar in mice expressing TRα1^R384C^ and in control littermates. Notwithstanding, adult mice expressing TRα1^R384C^ showed reduced motor performance on the Rotarod. T3 treatment during PND10-PND35 resulted in complete normalization of their locomotor behavior as adults. By contrast, T3 treatment in adults did not improve performance (2), indicating the existence of a specific time window for the action of TH/TRα1 signaling on brain development.

A series of mouse models mimicking human *THRA* mutations resulted in various degrees of alteration of the molecular functions of TRα1 (64). However, these mice exhibited little defects in cerebellar histology, the most notable defect being a slight reduction in the density of PV-expressing GABAergic interneurons in the molecular layer. The mild phenotype of these mice with frameshifts produced by genome editing contrasts with the severity of the phenotype of previously used *Thra* knock-in mice. Further investigations have revealed that the elimination of alternate splicing in these knock-in mice increased the expression level of the mutated TRα1 receptor and the severity of the phenotype (64).

In mice lacking both TH transporters, Mct8 and Oatp1c1 (*Mct8/Oatp1c1* DKO mice), TH signaling in the brain is significantly reduced and the arborization of the dendritic tree of Purkinje cells is significantly delayed, due a defect in intraneuronal transport of TH (65).

Finally, Amano et al. (66) have reported transient postnatal cerebellar defects, including alterations in granule cell migration and in Purkinje cell electrophysiological properties, in a mouse model of hypothyroidism, *Duoxa^-/-^* KO mice, which lack a dual oxidase that is essential for thyroid hormone synthesis. In particular, despite the fact that cerebellar histology returned to normal on postnatal day 25, motor coordination was still impaired at that age in *Duoxa^-/-^* mice, suggesting irreversible behavioral defects in these mice.

#### 3.2.2 Cerebral cortex

In 2008, Wallis et al. (63) provided a detailed histological study of different subtypes of cortical GABAergic interneurons in knock-in mice expressing TRα1^R384C^. Consistent with what had previously been described in hypothyroid rats, they found a developmental delay in the appearance of PV immunoreactive neurons in these mutant mice. An electrophysiological investigation of the PND19-PND21 cortex of mouse pups expressing TRα1^R384C^ revealed a 10-fold reduction in fast spiking neurons compared to controls. This was in line with the results of the immunohistochemical study, since many cortical PV immunoreactive cells are fast spiking neurons. At adult stages though, the density in PV neurons did not significantly differ between mutant and control mice (63, 67).

PV-expressing neurons were not the only GAABAergic neuron subtype found to be impacted by impaired TH signaling. Indeed, in mice expressing TRα1^R384C^, the density of calretinin-positive neurons in the cortex was significantly increased in adult mutant mice, compared to control mice. Regarding calbindin immunoreactivity, the authors described different results depending on the cortical layers under study: while mutant mice exhibited a lower density of calbindin-positive cells in layers II-III, there were no significant differences between mutant and control mice in layers IV-VI. The population of cortical somatostatin-positive neurons did not differ between mutant and control mice. Moreover, the total number of GABAergic cells in the cortex, as assessed by GAD67 immunoreactivity, did not differ significantly between mutant and control mice, indicating that the proliferation and migration of cortical GABAergic neuron precursors was unaffected by the mutation. As a whole, these results indicated that impaired TH/TRα1 signaling impacted the maturation of several populations of cortical GABAergic neurons, but the effects differed depending on the subtype of GABAergic neuron under study (63).

In an attempt to rescue the expression of PV in young mice expressing TRα1^R384C^, Wallis et al. (63) treated mutant mouse pups with TH between PND11 and PND13, but this failed to induce the expression of PV in the short term. By contrast, PV expression was restored in PND14 mice expressing TRα1^R384C^ that were exposed to high levels of TH from around birth. This suggests that TH/TRα1 signaling does not directly regulate PV expression, but rather influences the cell maturation process in a broader way.

Blocking TH entry into the brain also severely compromises the differentiation of cortical GABAergic interneurons. Indeed, Mayerl et al. (65) have recorded significantly reduced PV and Gad67 immunoreactivity (Gad67 being used as a marker of all GABAergic interneurons) in the somatosensory cortex of 12-day old and adult *Mct8/Oatp1c1* DKO mice, indicating that these defects were not transient, but permanent. In addition, they observed in the somatosensory cortex of adult, but not 12-day old, mice, a significant increase in the density of calretinin neurons. These results are congruent with those obtained in mice expressing TRα1^R384C^, in which the affinity of TRα1 for TH is significantly reduced (63). In agreement with the previous results, in *Mct8/Dio2* DKO mice, PV expression in cortical neurons was also found to be significantly reduced until adulthood (67). Moreover, several classes of GABAergic interneurons were found to be affected in mice expressing a mutated Mct8 transporter (P253L) mimicking a mutation found in human patients: in the cortex of these mice at adult stage, a decreased density of PV-, calbindin- and GAD65/67-positive neurons, as well as an increased density of calretinin-positive neurons, were reported (68).

#### 3.2.3 Hippocampus

*TRα1^-/-^* mice showed reduced PV perisomatic terminals on hippocampal CA1 pyramidal neurons, compared to controls (69). These structural defects were associated with poor performance in hippocampal-dependent behavioral tasks.

Likewise, in adult mice expressing TRα1^R384C^ dominant negative receptor, the number of PV-positive cell somata and the density of PV-positive terminals in the CA1 region of the hippocampus were found to be significantly reduced, compared to control mice (2). Fast-spiking PV-expressing interneurons are involved in the generation of rhythmic network oscillations in the gamma frequency range, which play an important role in higher processes in the brain, such as learning, memory, cognition and perception (70, 71). Extracellular field recordings from the stratum pyramidale in hippocampal slices (63) showed that the gamma oscillation frequency (20–80 Hz) was significantly lower in mutant mice expressing TRα1^R384C^, compared to controls, which was congruent with the reduced number of PV-expressing neurons (63). Moreover, hippocampal pyramidal neurons from mice expressing TRα1^R384C^ showed hypoexcitablility, compared to those of control mice (72). The notable impairments in the maturation of GABAergic neurons in knock-in mice expressing TRα1^R384C^ led the authors to suspect that these mice might be more susceptible to epilepsy than control mice. Unexpectedly, they were found to present a marked resistance to pentylenetetrazole-induced seizures, compared to control mice (72). Accordingly, pentylenetetrazole induced a significant increase in neuronal activity in the hippocampus of control mice, but not of mice expressing TRα1^R384C^. This phenotype was likely due to altered chloride homeostasis in principal neurons of mutant mice. In normal mouse neurodevelopment, GABAergic transmission is excitatory at early postnatal stages. During the second and third weeks of life, changes in the expression of chloride channels in principal neurons lead GABAergic transmission to switch from excitatory to inhibitory (73). Since Hadjab-Lallemend and colleagues (72) have found that mice expressing TRα1^R384C^ exhibited an imbalance in chloride channel subtypes in principal neurons, it is suspected that in these mice GABAergic transmission is maintained in an immature state, *i.e*. excitatory, until adulthood. Exposure to high levels of TH during both embryonic and postnatal developmental periods combined but not in adulthood, allowed to normalize the seizure behavior observed in these mutant mice (72).

The brain defects observed in mice expressing TRα1^R384C^ were also accompanied by significant changes in hippocampal-dependent behavior, indicative of increased anxiety and impaired memory (2). Interestingly, most of these behavioral defects, together with the structural defects in the hippocampus, could be reversed by exogenous administration of a high dose of TH for 12 days in adulthood. By contrast, the anxiety and memory defects observed in adulthood could not be prevented by an early TH treatment (between PND10 and PND35) (2). The latter results on mice expressing TRα1^R384C^ are at odds with the clinical observation that many of the defects induced by altered TH signaling on brain development are irreversible unless they are treated early in life. They underscore the complexity of TH action in the brain, and the necessity to get a better knowledge of the timely action of TH in different brain regions.

Intriguingly, a short-term treatment of knock-in mice expressing TRα1^R384C^ with a GABA receptor antagonist (pentylenetetrazol) rescued their memory performance, and this was accompanied by histological and electrophysiological changes reflecting an increase in the local excitatory drive in the CA1 region of the hippocampus (74).

In mice expressing a mutated Mct8 transporter (P253L), histological analysis of the hippocampus revealed defects in GABAergic interneuron populations that were similar to those previously described in the cortex: decreased density of PV-, calbindin- and GAD65/67-positive neurons, as well as increased density of calretinin-positive neurons (68).

#### 3.2.4 Hypothalamus

Mittag et al. (75, 76) have described a population of PV-expressing neurons in the mouse anterior hypothalamus, which requires prenatal signaling *via* both TRα1 and TRβ isoforms for proper development. These neurons are involved in the central autonomic control of blood pressure and heart rate, and are also temperature-sensitive. As hypothyroidism in humans is associated with bradycardia (77), it is conceivable that these effects are mediated by PV-expressing hypothalamic neurons. However, to our knowledge, there is no evidence that this population of PV-expressing neurons is GABAergic, as Laing et al. (78) have recently reported that anterior hypothalamic PV-expressing neurons in mice are glutamatergic.

### 3.3 Conditional mutant mouse models targeting specific cell types point to a direct effect of TH/TR signaling in developing GABAergic neurons

Rodent models of hypothyroidism as well as classical knock-in and KO mouse lines, as reviewed in the preceding paragraphs, have shed light on the complex influence of thyroid hormone on brain development, with GABAergic neurons appearing as particularly sensitive to impaired TH signaling. The advent of conditional mutagenesis, allowing to alter TH signaling in specific cell types, has allowed to get more insight into the brain cell types in which TH has a direct action.

*Thra^AMI^* allele encodes for a dominant-negative version of TRα1 (TRα1^L400R^), which is expressed only in cells where Cre recombinase is present (55). Ubiquitous expression of TRα1^L400R^ was shown to induce a severe phenotype, leading to death around the 3^rd^ or 4^th^ week of life (55). The same level of severity was observed when TRα1^L400R^ was expressed exclusively in brain cells (79). A detailed histological analysis of the cerebellum in these mice has revealed profound alterations in neuronal and glial differentiation, which were reminiscent of congenital hypothyroidism, including a strong reduction in the size and density of Purkinje cell arborization, a delay in GABAergic interneuron maturation, a delay in the migration of granule cell progenitors and abnormal Bergmann glia maturation (80). Crossing *Thra^AMI^* mice with mice expressing Cre in specific cerebellar cell types allowed to carry out a genetic dissection of the effects of TH in the developing cerebellum (81). The principal targets of TH in the cerebellum proved to be Purkinje cells, GABAergic interneurons, oligodendrocyte precursor cells and Bergmann glia (79, 82). Strikingly, the migration of granule cell precursors was altered when TH signaling was blocked specifically in Bergmann glia, or in Purkinje cells and GABAergic interneurons, but not in the least when TH signaling was blocked in granule cells themselves. Similar observations have been made in mice expressing a dominant-negative TRβ receptor in cerebellar Purkinje cells. Indeed, these mutant mice exhibited delayed Purkinje cell dendrite arborization, as well as delayed granule cell migration (83). Collectively, these results indicate that the defect in radial migration of granule cell precursors, which is a typical hallmark of the hypothyroid cerebellum, is not a cell-autonomous consequence of the lack of TH signaling, but rather results from an alteration of granule cell precursor environment (79).

Mice expressing a mutated dominant negative TRα1 receptor in all GABAergic neurons (either TRα1^L400R^ or TRα1^E395fs401X^) were found to present epileptic seizures as early as 11 days of age (3). At two weeks of age, the maturation of GABAergic neurons of different types (PV-, somatostatin-, NPY- or calretinin-expressing cells) appeared to be severely impaired, in the cerebellum as well as in the cortex, hippocampus and striatum. In particular, the density of PV-expressing neurons was drastically reduced in all these brain areas. Most of these mice died before the end of the 4^th^ week of life. The mice that survived until adulthood exhibited signs of hyperactivity and the defects in GABAergic neurons were still present. Notably, there was no normalization of PV expression over time (3). This was the first demonstration that TH signaling has a cell-autonomous effect influencing the maturation of GABAergic neurons, and that this developmental effect has lifelong consequences.

Mice expressing a dominant-negative TRβ receptor in cerebellar Purkinje cells were found to exhibit significant impairment in altered long-term synaptic plasticity at parallel fiber–Purkinje cell synapses in adulthood, even though there was no abnormality in the morphology or basal properties of these synapses at this age (84). These results stress the importance of TH action during neural development in establishing proper cerebellar function in adulthood, even if cerebellar morphology appears to be normal.

Conditional mutagenesis was also used to abolish TH transporter expression specifically in progenitors of PV interneurons [*Mct8 fl/fl; Oatp1c1 fl/fl; Nkx2.1Cre* mice (85)]. This induced a reduction in the density of PV+ interneurons, as well as an increase in the density of calretinin-positive neurons in the somatosensory cortex of 12-day old pups. These results clearly point to PV-expressing neurons as direct targets of TH signaling during development. However, cell numbers normalized in the adult conditional KO mice, whereas these changes were sustained at later time points when the same transporters were knocked out ubiquitously (*Mct8/Oatp1c1* DKO mice), indicating that the influence of TH on PV neuron maturation relies not only on cell-autonomous effects, but also on TH signaling in other cell types (85). As Sonic hedgehog (Shh) signaling pathway in the medial ganglionic eminence is known to play a key role in determining the fate of PV neuron progenitors (86), the level of activation of this pathway was assessed in ubiquitous *Mct8/Oatp1c1* DKO mice and in conditional *Mct8 fl/fl; Oatp1c1 fl/fl; Nkx2.1Cre* mice. At early stages of brain development, *i.e.* E12.5, it was found that Shh signaling was significantly reduced in the medial ganglionic eminence of *Mct8/Oatp1c1* DKO mice, but not in conditional *Mct8 fl/fl; Oatp1c1 fl/fl; Nkx2.1Cre* mice. In other words, Shh pathway in PV neuron progenitors was impacted when TH transporters were knocked out ubiquitously, but not when they were knocked out specifically in PV neuron progenitors. This indicates that non-cell autonomous mechanisms must relay the influence of TH on Shh signaling pathway in PV neuron progenitors of the medial ganglionic eminence (85).

As a conclusion, the current understanding is that what was initially found in the cerebellum also holds true in the rest of the brain: TH acts directly on a limited number of cell types, notably GABAergic neurons, but its influence propagates to other cell types through intercellular communication, notably *via* neurotrophins (79, 82).

## 4 Challenges in identifying TR target genes in developing GABAergic neurons

### 4.1 Identification of TR target genes in developing GABAergic neurons in rodents

Since RNA-seq has advantageously replaced microarray analysis, a growing number of datasets of gene expression linked to TH signaling has accumulated [reviews in (87) and (41)]. These results are theoretically suitable for identifying genes which are putative TR target genes in GABAergic neurons and identifying the molecular mechanisms that lead from TH stimulation to neuronal maturation. However, this remains a difficult task. To start with, although early studies have shown that a few genes, notably *Hr* and *Klf9*, are T3-responsive in many cell types (88, 89), more recent studies have mainly demonstrated that the repertoire of TH-responsive genes widely varies across cell types and brain areas (41). Thus, it finally appears that the overlap between sets of TR target genes in different types of cells might be limited. As regards developing GABAergic neurons, this implies that TH might not play the same role in cortical fast-spiking parvalbumin neurons, striatal medium spiny neurons or cerebellar Purkinje cells, to mention a few.

In spite of continuous advances in gene expression analysis techniques, identifying true TR target genes in developing GABAergic neurons remains challenging, for a number of reasons. One major issue is to handle the extreme cellular heterogeneity of the brain. As GABAergic neurons represent a minority of the cell population in most brain areas, the response to TH in GABAergic neurons is often masked by the response to TH in other cell types. The striatum is a favorable exception, as it is mainly populated by GABAergic medium spiny neurons (3, 90). For brain areas where GABAergic neurons are less abundant, RNA sequencing can be advantageously coupled to cell sorting in order to analyze gene expression levels in a specific cell type.

Several criteria should be fulfilled for a gene to be considered as a direct TR target gene. First, if one considers that TRs are essentially transcription activators, their target genes are expected to be down-regulated in the brain of hypothyroid mice or in the brain of mice carrying mutations that impair TH signaling (74, 91–93). Gene expression analyses in a variety of mouse models with impaired thyroid hormone signaling have confirmed that TR KO mice have an attenuated phenotype compared to hypothyroid mice, which is in agreement with a potent role of unliganded TRs in the repression of gene expression (13).

Second, when comparing gene expression levels between different conditions in a given brain region, one must take into account that cell composition may differ between conditions. In the analyses of mixed cell populations, like the whole cortex, whole striatum (3) or primary neuronal cell cultures prepared from fetal cortex (94) or from post-natal cerebellum (95), a decrease in the abundance of a GABAergic neuron-specific mRNA caused by hypothyroidism or by a genetic mutation is not sufficient to conclude that TH directly regulates the transcription of this gene within GABAergic neurons. An alternative explanation is that the long-term alteration of TH signaling has modified the composition of the cell population in hypothyroid/mutant mice, and that GABAergic neurons are under-represented in the brain area under study, when compared to control mice. A way to circumvent this problem is to analyze gene expression levels a few hours after treating mice with TH, and combine these results with those obtained in hypothyroid and mutant mouse groups. Considering genes that are upregulated shortly after TH treatment and downregulated in mice with impaired TH signaling tightens the analysis around potential TR target genes, while avoiding secondary effects due to tissue reorganization.

A third difficulty in determining the direct influence of TH/TR signaling on the transcription of a given gene in GABAergic neurons is to rule out effects that are downstream of TH signaling. Indeed, in many genetically modified animals as well as in models of pharmacologically-induced hypothyroidism, general growth and development are significantly affected, so that it is likely that there are additional factors, secondary to TH signaling disruption, that contribute to the neurological status and neuroanatomical integrity. Thus, changes in gene expression that are recorded in GABAergic neurons may be secondary to an extracellular event, such as induction of neurotrophin secretion by T3 by neighboring cells (96). Primary cell cultures constitute a way to reduce interactions between neighboring cell types (94, 95). One of the most powerful methods to investigate what is going on in a specific cell type *in vivo* relies on Cre/loxP recombination, which allows the mutation of specific genes in specific cell types. This approach was used to study gene expression in the striatum of *Thra^AMI/gn^* mice, in which the expression of the dominant-negative TRα1^L400R^ mutant receptor selectively abrogates response to TH in GABAergic neurons. As a whole, the putative direct TR target genes identified in that study did not highlight a specific pathway, but rather illustrated that TH signaling in GABAergic neurons is likely to affect a wide variety of functions such as cellular interactions, axon pathfinding or electrical and synaptic activity of the cell (3).

Finally, the cell-autonomous response of gene expression, as identified by RNA sequencing data from cell type-specific mutant mouse models, is not a full demonstration for a TR-mediated transactivation. Indeed, the effect of TH on a given gene can also be secondary to an intracellular event. For example, it can result from the TH-induced upregulation of a transcription activator. Although a time-course analysis following short-term TH treatment helps to recognize genuine TR target genes, one of the best current indications for a direct transcriptional activation relies on chromatin analysis. In the striatum, the expression of a tagged TRα1 expressed only in GABAergic neurons has allowed to address the occupancy of chromatin at a genome-wide scale. This has led to the conclusion that, although thousands of genomic sites are occupied by TRα1, the number of genes that are transcriptionally activated by the ligand-activated receptor is surprisingly small (3). Atac-seq analysis may be used to identify TH-induced changes in chromatin compaction, which indirectly inform of TR occupancy. One of the main advantages of this technique is that it can be efficiently implemented even when starting with small cell numbers (97).

Up to now, the analysis of TR target genes has failed to provide a unified picture of the influence of TH in GABAergic neurons. However, it is clear that many genes identified as TH-responsive in primary cultures of cortical neurons are related to the radial migration and terminal differentiation of cortical GABAergic interneurons (94). Gene expression analyses have also provided interesting working hypotheses, some of which have been tested in *in vitro* systems. Thus, it has been shown that a crosstalk between the signaling pathways mediated by TH on the one hand, and by αvβ3 integrin on the other hand, seems to play an important role in postnatal dendritic arborization of Purkinje cells (98). Another notable example is the demonstration that an up-regulation of *Klf9* by TH is a key event in the postnatal loss of the regenerative capacity that Purkinje cells display after axotomy (99).

When analyzing genome-wide datasets, a special attention has often been paid to the *Pvalb* gene, which encodes PV. Indeed, histological analyses have consistently shown a reduction in PV expression in rodent models with altered TH signaling (see section 3 in the present review). In a microarray analysis conducted in the cortex and striatum of 21-day-old hypothyroid mouse pups, *Pvalb* came out as one of the most strongly downregulated genes (13). Even subclinical hypothyroidism was shown to induce a large fold decrease in *Pvalb* mRNA expression in 14-day-old rats (100). Moreover, *Pvalb* mRNA levels in the cortex and striatum were significantly lower in *TRα1^-/-^* and *TRα1^-/-^ TRβ1^-/-^* mouse pups than in control mice. By contrast, *Pvalb* mRNA levels were not affected in *TRβ1^-/-^* mice, suggesting a predominant implication of TRα1 in mediating the effects of TH on PV expression (13). Finally, in *Thra^E403X/E403X^* mice, which express the first *Thra* mutation that was discovered in a patient, RNA-sequencing analysis has shown that genes such as *Flywch2*, *Pvalb*, and *Syt2*, which are preferentially expressed in PV-expressing neurons, were downregulated compared to control mice (93). As a conclusion, *Pvalb* expression is significantly reduced in mouse models with altered TH signaling, but up to now there is no convincing evidence that *Pvalb* is a direct target gene of TRs.

### 4.2 Insights from gene expression studies in the human brain

Even if the rodent brain is widely used as a model to decipher what is going on in the human brain, the brains of rodents and primates differ in several ways and studies in the human brain, when available, are extremely valuable to help translating the rodent data to the clinic. Notably, in the primate cortex, GABAergic neurons account for about 20% of the total neuron population, whereas in rodents, this percentage is about 15%. This difference is mainly due to an increase in the calretinin-expressing interneuron population. It is thought that the increased interneuron population is related to the increased associative functions and connectivity of the primate cortex, compared to the rodent cortex (101). Another major difference between the mouse and human brains relates to TH transporters in the blood-brain-barrier: in mice, both Mct8 and Oatp1c1 play a role in TH entry into the brain, whereas Oatp1c1 is not present in the human blood-brain-barrier. This explains why disruption of the *Mct8* gene in mice does not result in neurological impairment, while it has severe consequences in humans (101, 102).

Datamining in single cell RNA-seq studies was performed for the human fetal cortex at gestational weeks 16–18, equivalent to mid gestation in rodents (103). Although a similar analysis has not been performed in mice, it seems that the expression pattern of the main components of TH signaling is not the same in human and rodents. In particular, some cells of the human GABAergic lineage express *THRB* at this early stage, whereas it appears that *Thrb* expression is induced at later stages in the mouse brain (8). More precisely in humans, *THRB* expression is predominant in the subpopulation of GABAergic neuron progenitors migrating from the caudal ganglionic eminence and in calretinin-expressing interneurons that derive from these progenitors. This raises the interesting possibility that *THRB* mutations selectively alter calretinin-expressing GABAergic interneurons in the human cortex. Moreover, *SLC16A2* (encoding Mct8 transporter) and *THRA* show widespread expression in most human cortical cell types.

## 5 Concluding considerations

GABAergic neurons, and notably PV-expressing neurons, are a main target of TH signaling during brain development. Although the precise instrumental role of TH in these neurons remains elusive, a widely accepted working hypothesis considers that TH promotes the transition from the embryonic to adult pattern of gene expression in the brain (94, 104). For example, TH is involved in triggering the loss of axon regenerative capacity in Purkinje cells (99), such loss of axon regenerative capacity being a hallmark of brain maturation in rodents (105). At a wider scale, it is tempting to speculate that TH is involved in the regulation of critical periods of heightened plasticity in the brain (106). In agreement with such hypothesis, blocking TR signaling specifically in GABAergic neurons was found to significantly impair the development of perineuronal nets, which constitute a specialized extracellular matrix enwrapping mature PV-expressing neurons (3). Thus, TH signaling might trigger the setting-up of perineuronal nets, which stabilizes neuronal networks after taking into account the input from environmental stimuli (107). Such mechanisms are critical for proper brain development.

GABAergic neurons are fundamental for maintaining the balance between excitation and inhibition throughout the brain (108). In particular, PV-expressing neurons play key roles in the coordination of neuronal networks and associated oscillations (70). Thus, as impaired TH signaling during brain development significantly affects GABAergic neurons in general, and PV-expressing neurons in particular, this may account for many of the neurological disorders seen in patients with impaired TH signaling. Indeed, as was previously mentioned, studies that were carried out in in the last ten years have revealed that patients with *THRA* mutations display a high risk of epilepsy and ASD (Section 2 of the present review).

Besides being highly sensitive to altered TH signaling, PV-expressing neurons appear as an important node in many neurodevelopmental disorders, including ASD and epilepsy. ASD is a multifactorial neurodevelopmental disorder that encompasses a complex and heterogeneous set of traits. One unifying explanation for the complexity of ASD may lie in the disruption of the balance between excitatory and inhibitory circuits during critical periods of development (109), which echoes our current understanding of TH action in GABAergic circuits. Moreover, post-mortem studies of the cerebral cortex of ASD patients have revealed that the number of PV-expressing interneurons was decreased, and that *Pvalb* was the most strongly downregulated gene, compared to control patients (110, 111). Finally, Berbel and collaborators (112) have highlighted that brain morphological changes observed in mouse models of developmental hypothyroidism, such as alterations in cortical lamination, high neuronal density in several hippocampal layers, poor differentiation of the gray-white matter boundary or neuronal heterotopias, resembled the brain lesions of children with autism. The same authors noted that a large number of genes that have been found to be TH-regulated at the transcriptional level in rodent cerebral cortex have also been found to be mutated in ASD patients (112, 113).

As for ASD, epilepsy encompasses a group of multifactorial diseases, suggesting that diverse genetic or environmental insults may impair common pathways, leading in the end to symptoms of epilepsy. Again, PV-expressing neurons might be at the crossroads of these common pathways. Indeed, impaired development or function of PV-expressing interneurons has been associated with some genetic forms of epilepsy in humans (114). As a consequence, PV-expressing neurons have been identified as a critical target of therapeutic approaches in epilepsy (115).

Collectively, the convergence of symptoms between hypothyroid, epileptic and ASD patients suggests that common pathways involving PV-expressing neurons might underlie these pathologies of brain development. Such convergence might partly be linked to comorbidities that contribute independently to the overt pathology, but in any case, improving our understanding of the role of TH in the development of the GABAergic system, notably in PV-expressing neurons, should help make progress in the prevention and treatment of several neurological disorders. In addition, deciphering the network of TH target genes in the brain may help detect pharmacological or chemical agents that are likely to disrupt TH signaling, and give an insight on subtle neurological insults that may result from exposure to such TH system-disrupting chemicals (116). However, there is still a long way to go before we understand the precise molecular mechanisms underlying TH action in the brain. Notably, the huge diversity of GABAergic neurons (117) makes it difficult to depict a unified view of the mechanisms of action of TH in these neurons. It is hoped that the advent of single-cell RNA sequencing (118) and of spatio-temporal transcriptomics (119) will help untangling the role of TH signaling in each GABAergic neuron subtype, in each brain region, at all developmental stages.

## Author contributions

FF: Writing – original draft, Writing – review and editing. SR: Writing – original draft, Writing – review and editing. JR: Writing – review and editing.
